# Declining Sex Ratio in a First Nation Community

**DOI:** 10.1289/ehp.8479

**Published:** 2005-08-17

**Authors:** Constanze A. Mackenzie, Ada Lockridge, Margaret Keith

**Affiliations:** 1 Faculty of Medicine, University of Ottawa, Ottawa, Ontario, Canada; 2 Aamjiwnaang Environment Committee, Aamjiwnaang, Ontario, Canada; 3 Occupational Health Clinics for Ontario Workers Sarnia-Lambton, Pt. Edward, Ontario, Canada

**Keywords:** community-based, endocrine disruption, environmental exposure, First Nation, sex ratio

## Abstract

Members of the Aamjiwnaang First Nation community near Sarnia, Ontario, Canada, voiced concerns that there appeared to be fewer male children in their community in recent years. In response to these concerns, we assessed the sex ratio (proportion of male births) of the Aamjiwnaang First Nation over the period 1984–2003 as part of a community-based participatory research project. The trend in the proportion of male live births of the Aamjiwnaang First Nation has been declining continuously from the early 1990s to 2003, from an apparently stable sex ratio prior to this time. The proportion of male births (*m*) showed a statistically significant decline over the most recent 10-year period (1994–2003) (*m* = 0.412, *p* = 0.008) with the most pronounced decrease observed during the most recent 5 years (1999–2003) (*m* = 0.348, *p* = 0.006). Numerous factors have been associated with a decrease in the proportion of male births in a population, including a number of environmental and occupational chemical exposures. This community is located within the Great Lakes St. Clair River Area of Concern and is situated immediately adjacent to several large petrochemical, polymer, and chemical industrial plants. Although there are several potential factors that could be contributing to the observed decrease in sex ratio of the Aamjiwnaang First Nation, the close proximity of this community to a large aggregation of industries and potential exposures to compounds that may influence sex ratios warrants further assessment into the types of chemical exposures for this population. A community health survey is currently under way to gather more information about the health of the Aamjiwnaang community and to provide additional information about the factors that could be contributing to the observed decrease in the proportion of male births in recent years.

There is increasing evidence that the human live birth sex ratio can be altered by a number of environmental and occupational chemical exposures. For example, lower proportions of male offspring have been observed in populations exposed to dioxin (Mocarelli et al. 2000), mercury (Sakamoto et al. 2001), pesticides (Garry et al. 2003; Goldsmith 1997; Jarrell et al. 2002), polychlorinated biphenyls (PCBs) (del Rio Gomez et al. 2002; Weisskopf et al. 2003), and parental smoking (Fukuda et al. 2002). It has been hypothesized that some of these environmental and occupational chemicals may act as endocrine-disrupting compounds (EDCs), influencing the sex ratio by changing the hormonal milieu of the parents (James 1996), or by inducing sex-specific mortality *in utero* (Sakamoto et al. 2001).

The birth sex ratio (male:female) of a population is often reported as the male proportion (*m* = number of male births divided by the total of all births). Worldwide, the human live birth sex ratio is remarkably constant, ranging between 102 and 108 male to 100 female live births (*m* = 0.504–0.519) (Bartleby.com 2003). In Canada, the sex ratio is generally reported to be 105:100 (M:F) (*m* = 0.512) (Allan et al. 1997). Although the sex of the human embryo is genetically controlled and determined at the time of conception, there is evidence that the sex ratio can be partially influenced by both endogenous and exogenous factors. Endogenous parental hormone concentrations of gonadotropins and/or testosterone at the time of conception are suspected to play a role in determining the sex of offspring (James 2004). Exogenous factors such as stress, parental disease, and exposure to certain chemicals appear to have some influence on the live birth sex ratio and may act by altering the parental hormone status (James 2004).

Sex ratios have been suggested as a non-invasive monitor of the reproductive health of a population (Davis et al. 1998; James 1997a). Changes in the sex ratio have been used to assess the reproduction of populations with demonstrated exposures to EDCs (Mocarelli et al. 2000), as well as in communities near hazardous chemical sites (Williams et al. 1995). Altered live birth sex ratios may also be a useful indicator of public health, in that they reflect death at earlier stages of development than traditional indicators such as perinatal and infant mortality (Williams et al. 1995).

## Materials and Methods

We assessed the live birth sex ratios for the Aamjiwnaang First Nation community in Ontario, Canada, in response to concerns voiced by members of the community regarding the perception of fewer male children in recent years. This birth sex ratio assessment was part of a broader community-based investigation undertaken by the Aamjiwnaang in collaboration with the Occupational Health Clinics for Ontario Workers (OHCOW) along with scientific consultants, professionals, and students from a wide range of disciplines. The exploration included such quantitative measurements as soil, sediment, wildlife, fish, and air sampling, along with a door-to-door health survey and interviews. In keeping with the principles of community-based participatory research (Hall 1979, 2003; Hagey 1997; Hills and Mullett 2000; Keith and Brophy 2004), the community itself has been involved in all major decision-making about the direction of the research and has participated in much of the data collection.

### Aamjiwnaang First Nation.

The Chippewas of Aamjiwnaang have approximately 850 band members residing on the Aamjiwnaang reserve land (Information Management Branch Department of Indian Affairs and Northern Development 2001). This reserve is located within the area identified as the St. Clair River Area of Concern by the Canada–U.S. Great Lakes International Joint Commission (Environment Canada 2005) and is situated immediately adjacent to the Sarnia-Lambton Chemical Valley—one of Canada’s largest concentrations of industry. The reserve is surrounded by several large petrochemical, polymer, and chemical industrial plants. The community provided informed consent and assistance to collect live birth sex ratio data from the Department of Indian and Northern Affairs database (Indian and Northern Affairs Canada, Ottawa, Ontario, Canada) for the period 1984–2003 (representing the full length of record). Births and deaths of members of the Aamjiwnaang First Nation are reported on a monthly basis to the Department of Indian and Northern Affairs by the Aamjiwnaang Lands and Memberships clerk.

### Statistical analysis.

We calculated the proportion of male births for the Aamjiwnaang community by dividing the number of male live births by the total of all live births for each year 1984–2003. We used linear regression to examine the trend in the proportion of male births over time. Based on the data, we produced two linear regression lines to estimate a point in time where slopes of the regression lines deviate and the trend in the proportion of male births begins to decline. We then calculated the proportion of live male births for both 5- and 10-year intervals over the duration of the study period and compared these data to the expected proportion of males for Canada (*m* = 0.512), as well as a “control” First Nation community (*m* = 0.520) using Pearson’s chi-square analysis. The “control” community sex ratio was calculated from comparable data for a genetically similar, yet geographically distinct, Chippewa First Nation band that has requested to remain anonymous. Because the male proportion in the “control” community (*m* = 0.520) was not statistically different from the expected Canadian male proportion (χ^2^ = 0.098, df = 1, *p* = 0.754), all analyses shown were performed using the Canadian male proportion (*m* = 0.512) as the expected value.

## Results

### Altered sex ratios.

**Figure 1:**
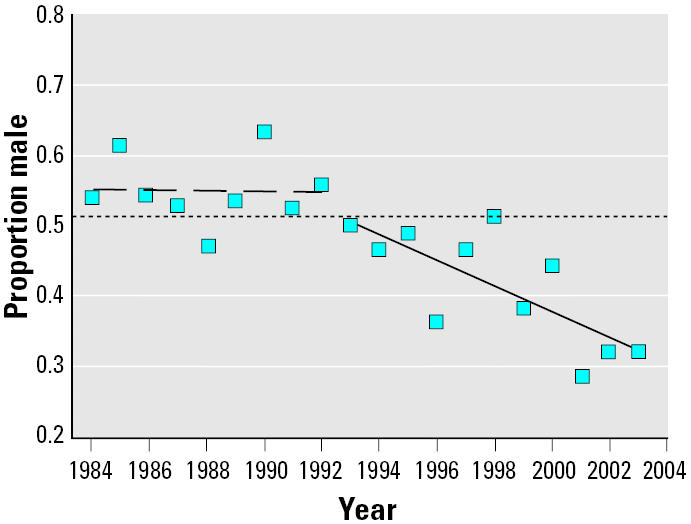
Proportion of live male births (male live births/total live births) for Aamjiwnaang First Nation 1984–2003. The dotted line is the expected male proportion for Canada (0.512). The dashed line is the linear regression line for the period 1984–1992; *r*^2^ = 0.000; slope not significantly different from zero (*p* = 0.990). The solid line is the linear regression line for the period 1993–2003; *r*^2^ = 0.547; statistically significant deviation of slope from zero (*p* = 0.009).

Examination of the proportion of male births for the Aamjiwnaang community over the study period 1984–2003 (Figure 1) shows that the proportion of male births appears to be relatively stable for the period 1984–1992; linear regression (*r*^2^ = 0.000) shows a slope not significantly different from zero (*p* = 0.990). A second linear regression for the period 1993–2003 (*r*^2^ = 0.547) shows a declining trend in the proportion of male births with a statistically significant deviation of slope from zero (*p* = 0.009).

When the sex ratio data were categorized into 5- and 10-year periods, we found a highly significant decrease in the proportion of male live births (*m* = 0.348, χ^2^ = 7.472, df = 1, *p* = 0.006) for the Aamjiwnaang community during the most recent 5-year interval (1999–2003) compared with the expected sex ratio (Table 1). We also observed a statistically significant decrease in the proportion of male births when the data were categorized into 10-year intervals over the period 1994–2003 (*m* = 0.412, χ^2^ = 7.100, df = 1, *p* = 0.008) (Table 1).

## Discussion

### Evidence of chemical exposures influencing sex ratios.

Normal variation in sex ratio can be expected in any population, especially with a small sample size; however, the extent of the sex ratio deviation for Aamjiwnaang appears to be outside the range of normal. Following relatively stable sex ratios from 1984–1992, there was a significant decline in the proportion of male live births for the period 1993–2003 (Figure 1). The continuing reduction in the proportion of male births is most apparent in the most recent 5-year period (1999–2003, m = 0.348; Table 1), indicating that there may be an ongoing process manifesting as a reduction in sex ratio starting in the early to mid-1990s. Previous studies have demonstrated that populations exposed to environmental contaminants such as endocrine disruptors, either through their close proximity to industrial plants or through other sources such as food, can have significant changes in the reproductive ability of the community, including the sex ratio. Table 2 summarizes some findings on the influence of environmental and occupational exposures on sex ratios.

There has been speculation that declining trends in the proportion of male births during the later part of the 20th century in industrialized countries including Canada, the United States (Allan et al. 1997), Sweden, Germany, Finland, Denmark, and the Netherlands (Davis et al. 1998) could be attributed to environmental contaminants and endocrine disruption. Conversely, other studies have shown increases in sex ratio (Lancaster and Day 1998), or no change (Grech et al. 2003), and it is unlikely that a single mechanism can account for the changes observed in any one country, given the scale of these studies (James 1998). However, changes in sex ratios of small populations can be used more reliably as a sentinel indicator of altered reproduction, especially when there is a documented exposure to environmental or occupational chemicals (James 1998).

There are several possible routes of exposure to chemicals that may affect the reproductive ability of a community. Populations can be exposed to contaminants through industrial accidents such as in Seveso, Italy, where young men exposed to high concentrations of dioxins sired significantly more female children than male (Mocarelli et al. 2000). Several studies have examined the sex ratios of communities exposed to different types of air pollution with conflicting results: a decrease in sex ratio was observed for residential areas exposed to air pollution from local incinerators (Williams et al. 1992); an increase in sex ratios was observed in areas close to a steel foundry (Lloyd et al. 1984, 1985), communities close to natural gas (Saadat et al. 2002) and petrochemical industry (Yang et al. 2000b); and no effect on sex ratio was observed for general air pollution (Williams et al. 1995) and in another (less powerful) study of municipalities close to a petroleum refinery plant (Yang et al. 2000a). Other routes of exposure to contaminants such as PCBs include food sources. Decreased sex ratios have been associated with maternal consumption of Great Lakes fish (Weisskopf et al. 2003) and fish contaminated with methylmercury (Sakamoto et al. 2001). To make matters more complicated, paternal Great Lakes fish consumption appears to increase the offspring sex ratio (Karmaus et al. 2002).

Although there is mounting evidence that environmental and occupational exposures to contaminants can affect sex ratios, the results to date can be difficult to interpret because of conflicting results and the number of variables that appear to be involved. The effect of a chemical exposure on a population’s sex ratio appears to depend on a number of factors, including parental age at the time of exposure, total exposure level, and whether it is a maternal or paternal exposure (Table 2). For example, similar exposures in men and women may have different effects on the sex of offspring, as observed with PCBs (Table 2).

Based on an assessment algorithm used by Jarrell (2002), there is reasonably strong evidence linking reduced sex ratios and environmental exposures of dioxin, dibromochloropropane, and hexachlorobenzene (HCB). Although the mechanism of action of these compounds on sex ratio is not entirely clear, both dioxin and HCB bind to the aryl hydrocarbon receptor and may alter sex ratios by changing the hormonal status of the parents. HCB is also associated with pregnancy loss in women (Jarrell et al. 1998). Similarly, other compounds such as methylmercury appear to increase the number of spontaneous abortions and stillbirths in exposed populations, ultimately altering the sex ratio of the surviving offspring (Sakamoto et al. 2001). The overall effects of other compounds such as PCBs on sex ratios will continue to be clarified with further research.

A 1996 assessment of soil and sediment contaminants on Aamjiwnaang reserve land has identified high concentrations [many exceeding sediment guidelines of the Ontario Ministry of the Environment and Energy (2004)] of several contaminants including PCBs, HCB, mirex, polycyclic aromatic hydrocarbons (PAHs), and metals (copper, nickel, lead, mercury, arsenic, chromium, manganese, iron) (Leadley and Haffner 1996). Pollutants released by the petrochemical industry surrounding the Aamjiwnaang reserve, as reported by the National Pollutant Release Inventory (Environment Canada 2002), are too numerous to name in entirety but include volatile organic compounds, ethylene, phthalates, dioxins and furans, HCB, vinyl chloride, PAHs, ammonia, acrylonitrile, and metals (nickel, mercury, lead, cadmium, zinc, manganese). Because of the close proximity of this community to the large aggregation of petrochemical industry and potential exposures to compounds with known effects on sex ratios, further investigations into the types and routes of chemical exposures (air, water, food, soil, and sediment) are warranted for this population.

### Evidence of altered wildlife reproduction in the area.

A large body of literature has accumulated detailing the adverse effects of EDCs on the reproductive ability and sexual development of fish, amphibians, reptiles, and birds. Numerous studies indicate that wildlife populations in the Great Lakes area are being adversely affected by the level of contamination, and that evidence from wildlife research could be used as a sentinel for human health effects (Fox 2001). In the Great Lakes area close to the Aamjiwnaang reserve, fish with intersex gonads (both male and female) have been reported in Lake St. Clair (Kavanagh et al. 2004). There is also ongoing research in the St. Clair River Area of Concern region of the Great Lakes that is documenting reduced hatching success and altered sexual development in turtles as well as changes in the sex ratios of birds (Environment Canada Canadian Wildlife Service 2003).

### Other population factors influencing sex ratio.

Numerous biological and environmental factors appear to have a minor influence on sex ratios, including parental age, parity, birth order, coital rates, infertility, maternal nutrition (James 1996, 2004), illness such as insulin-dependent diabetes mellitus (Rjasanowski et al. 1998), stress, war (Ansari-Lari and Saadat 2002), and selective reproductive practices (Allahbadia 2002). These influences on sex ratio are generally considered to play a small role, but cannot be ruled out completely without additional information about the study population.

One study has looked at the influence of race on sex ratios with North American Indian couples having slightly higher sex ratios (more boys) than Caucasian couples (Khoury et al. 1984). Comparison of the “control” Chippewa First Nation community to the average sex ratio for Canada showed no significant difference between the two populations, indicating that race is likely not playing a role in the observed altered sex ratio of the Aamjiwnaang community.

### Future studies.

The Department of Indian and Northern Affairs database is the most accurate source of information for First Nations vital statistics in Canada. However, there are some limitations to this database because it potentially includes births of band members that are not residing on reserve land and does not provide additional information about, for example, parental age, parity, or stillbirths. A community health survey is under way to explore a broad range of health concerns among the residents of the Aamjiwnaang reserve, as well as to gather information on covariates that may influence live birth sex ratio, including parental age, length of residency in the community, sex of stillbirths, and lifestyle factors such as parental smoking.

The initial assessment of the sex ratios of the Aamjiwnaang community over the 20-year period 1984–2003 presented here indicates that there is a significant ongoing decrease in the proportion of male live births beginning in the early 1990s and continuing to the end of the study period 2003. Although several potential factors may be contributing to the observed decrease in sex ratio, the close proximity of this community to the large aggregation of petrochemical industry and potential exposures to compounds that may influence sex ratios warrants further assessment into the types of chemical exposures for this population. Because of the complexities of any population exposure, causality between a single compound and adverse effect is always difficult to assess. It is possible that the Aamjiwnaang community has had multiple chemical exposures over the years that may be contributing to the overall picture of a reduced sex ratio. Further assessment must include the identification of exposures to compounds that may already be associated with adverse effects on sex ratio, determination that the timing of exposure is appropriate for the observed change in sex ratio, and elimination of other potential influences on sex ratio.

## Figures and Tables

**Table 1:** Total live births, proportion of live male births (male live births/total live births), χ^2^, and *p*-value for Aamjiwnaang First Nation 1984–2003 arranged in 5- and 10-year periods.

Period	Total live births	Proportion male births	χ^2a^	*p*-Value
5-Year				
1984–1988	173	0.538	0.185	0.667
1989–1993	185	0.551	0.532	0.466
1994–1998	215	0.451	1.574	0.210
1999–2003	132	0.348	7.472	0.006*
10-Year				
1984–1993	358	0.545	0.807	0.369
1994–2003	347	0.412	7.100	0.008*

a Chi-square was performed using an expected male proportion equal to 0.512; df = 1.

*Highly significant statistical deviation (p < 0.01) from the expected proportion of males using Chi-square analysis.

**Table 2:** The influence of environmental and occupational exposures on sex ratio.

Exposure type	Decreased sex ratio (fewer boys)	Increased sex ratio (fewer girls)	No effect
Dioxin	Paternal environmental exposure postindustrial accident (Mocarelli et al. 1996, 2000) Paternal exposure as pesticide producers (Ryan et al. 2002)		Paternal occupational exposure (Schnorr et al. 2001)
PCBs	Paternal consumption of rice oil contaminated with PCBs at < 20 years of age (del Rio Gomez et al. 2002) Maternal exposure to PCBs in Great Lakes fish (Weisskopf et al. 2003)	Paternal exposure to PCBs in Great Lakes fish eaters (Karmaus et al. 2002)	Consumption of rice oil contaminated with PCBs and PCDFs (Yoshimura et al. 2001)
Pesticides	Paternal exposure to nematocide DBCP (Goldsmith 1997) Pesticide applicators (Garry et al. 2003) HCB exposure (Jarrell et al. 2002)		Maternal exposure to HCB (Jarrell et al. 1998)
Methylmercury	Maternal exposure to methylmercury-contaminated fish (Sakamoto et al. 2001)		
Petroleum		Municipalities exposed to petrochemical air pollution (Yang et al. 2000b) Natural gas exposure (Saadat et al. 2002)	Municipalities adjacent to a petroleum refinery plant (Yang et al. 2000a)
Air pollution	Air pollution from incinerators (Williams et al. 1992)	Air pollution from local steel foundry (Lloyd et al. 1984, 1985)	General air pollution (Williams et al. 1995)
Radiation	Maternal exposure to non-ionizing radiation (electromagnetic radiation, strong static) and paternal exposure to high voltage (James 1997b)	Paternal occupational exposure to ionizing radiation (Dickinson et al. 1996)	Background ionizing radiation (Saadat 2003)
Occupation	Paternal exposure carbon type setters (Milham 1993)		
Infertility treatment	Maternal exposure to clomiphene citrate (Jarrell 2002)		
Lifestyle	Parental smoking (Fukuda et al. 2002)		

Abbreviations: DBCP, dibromochloropropane; PCDFs, polychlorinated dibenzofurans.
